# Dentin Phosphophoryn-Derived Peptide Promotes Odontoblast Differentiation In Vitro and Dentin Regeneration In Vivo

**DOI:** 10.3390/ma14040874

**Published:** 2021-02-12

**Authors:** Bayarchimeg Altankhishig, Mohammad Ali Akbor Polan, Youjing Qiu, Md Riasat Hasan, Takashi Saito

**Affiliations:** 1 Division of Clinical Cariology and Endodontology, Department of Oral Rehabilitation, School of Dentistry, Health Sciences University of Hokkaido, Hokkaido 061-0293, Japan; bayarchimeg@hoku-iryo-u.ac.jp (B.A.); qiuyoujing@hoku-iryo-u.ac.jp (Y.Q.); 2 Department of Children Preventive and Community Dentistry, Dhaka Dental College, Dhaka 1206, Bangladesh; ali_polan@yahoo.com

**Keywords:** dentistry, phosphophoryn-derived peptide, odontoblast differentiation, dentin regeneration, direct pulp capping, reparative dentin formation

## Abstract

The purpose of the present study was to investigate the effect of a peptide (i.e., SESDNNSSSRGDASYNSDES) derived from dentin phosphophoryn (DPP) with arginine-glycine-aspartic acid (RGD) motifs on odontoblast differentiation in vitro and to compare it with calcium hydroxide—a material used conventionally for vital pulp therapy—in terms of reparative dentin formation and pulp inflammation in vivo. Alkaline phosphatase activity assay and alizarin red S staining were performed to evaluate odontoblast-differentiation in cell culturing experiments. To observe the reparative dentin formation and pulp inflammation animal experiment was performed and examined by histological methods. The difference between the experimental group and the control group was analyzed statistically using a one-way ANOVA test. The results revealed that the DPP-derived RGD-containing peptide triggered odontoblast differentiation and mineralization in vitro. In rats undergoing direct pulp capping, the DPP-derived RGD-containing peptide was found to induce intensively formed reparative dentin with high compactness at week 4. On histological and morphometrical examinations, a smaller degree of pulpitis was observed in the specimens treated with the peptide than in those treated with calcium hydroxide. This study suggests that the DPP-derived RGD-containing peptide is a biocompatible, biodegradable and bioactive material for dentin regeneration.

## 1. Introduction

Tissue engineering and regenerative medicine have resulted in significant improvements in the treatment of diseases and defects over the past decades [1]. The current trends in tissue engineering are toward tissue regeneration and accordingly require the development and improvement of biomaterials. In recent years, numerous investigations in the field of tissue engineering have used extracellular matrices for inducing preferred cellular differentiation toward specific directions [2,3,4,5]. Extracellular matrices function as critical regulators of cell adhesion, migration, proliferation and differentiation during tissue repair. Moreover, they can be used as biocompatible, biodegradable materials because of their biological nature and safe application in the human body [6].

The major challenge in the use of tissue engineering for therapeutic dentistry involves inducing tooth regeneration as well as bone regeneration [7]. Dentin phosphophoryn (DPP) is the most dominant noncollagenous protein synthesized by odontoblasts in the dentin extracellular matrix [8]. It is a cleavage product of dentin sialophosphoprotein (DSPP) that belongs to small integrin-binding ligand N-linked glycoproteins (SIBLINGs)—a family that also includes bone sialoprotein (BSP), osteopontin (OPN), dentin matrix protein 1 (DMP-1) and matrix extracellular phosphoglycoprotein (MEPE), all of which contain arginine-glycine-aspartic acid (RGD) motifs [9,10]. Moreover, it comprises multiple repeats of the tripeptide aspartate-serine-serine (DSS) with highly (over 90%) phosphorylated serine residues that render it a high net negative charge, thereby facilitating binding to calcium ions [10].

Two types of DPP—free DPP (electrostatically bound to collagen fibrils) and immobilized DPP (covalently cross-linked with collagen fibrils), exist in the dentin extracellular matrix [11]. Some researchers have demonstrated the role of DPP in dentin formation. Moreover, DPP immobilized on type I collagen fibrils initiates hydroxyapatite formation in vitro [12]. Besides its role in mineralization, DPP immobilized on cell culture plates has been shown to induce proliferation, differentiation and mineralization of odontoblast-like cells [13,14]. Moreover, in a study of dentin regeneration in rats, DPP immobilized on type I collagen fibrils appeared to promote reparative dentin formation to a greater extent compared with conventionally used calcium hydroxide for vital pulp therapy [15]. These findings were attributed to the highly negative charge of DPP induced by repeated Asp-PSer-PSer and N-terminal RGD domains, resulting in signal transduction via binding to integrin α_v_β_3_ and α_4_β_1_ on cell surfaces [14,16].

However, other studies have revealed that free DPP in solution inhibited apatite formation in vitro [17,18]. This failure was shown to result from the self-association of DPP in the solution, including calcium and phosphate, followed by the suppression of the conversion of the sequestered calcium phosphate clusters that bind to the phosphate esters of repeated Asp-PSer-PSer domains, to a crystalline form of hydroxyapatite [19]. Nevertheless, the potential effect of the N-terminal RGD domain of free DPP on odontoblast differentiation and dentin regeneration remains less understood.

In the present study, we aimed to examine the effects of a DPP-derived RGD-containing peptide (SESDNNSSSRGDASYNSDES) free in solution on odontoblast differentiation in vitro and on reparative dentin formation after implantation in artificially exposed rat dental pulps and to investigate whether it could be employed as a useful biomaterial for deep dental caries treatment.

## 2. Materials and Methods

### 2.1. DPP-Derived RGD-Containing Peptide Synthesis

An RGD-containing peptide (SESDNNSSSRGDASYNSDES) derived from the human DPP amino acid sequence was custom synthesized by Invitrogen with a final purity of over 95% [13]. Then, the peptide was dissolved in distilled water (dH_2_O, Invitrogen, Carlsbad, CA, USA) and was subsequently diluted in Dulbecco’s modified Eagle’s medium (DMEM) (Sigma-Aldrich, St. Louis, MO, USA) to obtain the desired final concentration.

### 2.2. Cell Culture

Immortalized rat odontoblast-like MDPC-23 cells were generously provided by Professor Jaques E. Nör from the University of Michigan. The cells were cultured at initial densities of 5 × 10^4^ cells in 35-mm dishes, 1 × 10^5^ cells in 60-mm dishes and 1 × 10^4^ cells per well in 24-well plates (Corning, NY, USA). These cells were grown in DMEM supplemented with 10% fetal bovine serum (Sigma-Aldrich, St. Louis, MO, USA) at 37 °C in a humidified atmosphere of 5% CO_2_ and 95% air. Cells from passages 14 to 20 were used in this study [13].

### 2.3. Cell Morphology Observation and Cell Number Determination

Cells were incubated in the basic cell culture medium described above with 1 ng/mL of the DPP-derived RGD-containing peptide and were then visualized at days 1, 2 and 4 under phase-contrast microscopy (Olympus 1X70, Tokyo, Japan). The number of cells on the dish was counted from day 1 to day 10 to evaluate the effect of the peptide on cell growth and proliferation. The detached cells using TrypLE^TM^ express (Gibco, NY, USA) were counted using a hemocytometer.

### 2.4. Alkaline Phosphatase (ALP) Activity Assay

The cells were incubated in the basic cell culture medium described above with 1 μg/mL of the DPP-derived RGD-containing peptide and were subsequently collected and lysed with 0.1% Triton-X-100 (Sigma-Aldrich, St. Louis, MO, USA) in distilled water at day 7. The lysates were sonicated for 10 min on ice and then centrifuged at 12,000 rpm for 15 min at 4 °C. Thereafter, the supernatant was extracted and assayed for ALP activity (Wako, Osaka, Japan) and protein quantification (Thermo Fisher Scientific, Waltham, MA, USA). One unit of enzyme activity was defined as the release of 1 nmol of *p*-Nitrophenol per minute at pH 9.8 (37 °C). The relative activity was determined as follows: units/μg protein = Activity (units/μL)/Protein concentration (μg/μL). Absorbance was determined using a microplate reader (Bio-Rad, Hercules, CA, USA) at 405 nm and 570 nm for ALP activity assay and protein quantification, respectively.

### 2.5. Alizarin Red S Staining

MDPC-23 cells were grown on 24-well plates in the basic culture medium with 1 ng/mL of the DPP-derived RGD-containing peptide for eight days. The cell monolayer was fixed with 10% neutral formalin solution (060-01667, Wako, Osaka, Japan) for 20 min and stained with 1% alizarin red S solution (pH = 4.1; 011-01192, Wako, Osaka, Japan) in dark for 10 min in an incubator at 37 °C [13]. The mineralized nodules were photographed and counted using ImageJ software 1.44 (Wayne Rashand, Bethesda, MD, USA).

### 2.6. Surgical Procedure in Animal Experiments

Animal procedures were performed in accordance with the guidelines of the Animal Care Committee of the Health Sciences University of Hokkaido (approval no: 19-093). A total of eight male Wistar rats (8-week-old; Hokudo, Sapporo, Japan) were anesthetized with an intraperitoneal injection of pentobarbital (40 mg/kg). Both sides of the maxillary first molar were used in this experiment. Total four teeth (*n* = 4) were used in each group of the experiment. The vital pulp tissue of the maxillary first molar was exposed using a sterile, round, steel bur and was subsequently treated with 10% sodium hypochlorite and 3% hydrogen peroxide. Then, bleeding was controlled with sterile cotton pellets. Thereafter, the exposed pulp tissue was covered with 300 μg of the DPP-derived RGD-containing peptide (RGD group). Multi-Cal (Pulpdent, Watertown, MA, USA), the conventional agent for vital pulp therapy, was used as the control (Ca group). Next, each cavity was sealed with HY-Bond GlasIonomer CX (Shofu, Koto, Japan) and filled with Clearfil S-3 Bond (Kuraray, Tokyo, Japan) and Unifil flow (GC, Tokyo, Japan).

### 2.7. Histological Examination

At two and four weeks after surgery, the rats were anesthetized with pentobarbital. After perfusion with 4% paraformaldehyde in 0.1 mol/L phosphate buffer (pH 7.4), all experimental teeth and the surrounding alveolar bone were removed. The specimens were immersed in the same fixative for a further day. Specimens were then decalcified in 10% ethylenediaminetetraacetic acid (EDTA) (Nacalai Tesque, Tokyo, Japan) for 15 days at 4 °C, dehydrated with increasing concentrations of ethanol and then embedded in paraffin. Serial sections of 4 μm thickness were placed on silane-coated slides, deparaffinized with clear advantage (POL24770; Polysciences Inc., Warrington, DC, USA) and rehydrated with decreasing concentrations of ethanol in distilled water. Sections were stained with hematoxylin and eosin (HE) (Nacalai Tesque, Tokyo, Japan) and observed by light microscope (Eclipse E400, Nikon, Tokyo, Japan).

### 2.8. Morphometrical Analysis of the Formed Reparative Dentin and Evaluation of Pulp Inflammation

The formation rate of reparative dentin, that is, the relative area of the reparative dentin formed in the cavity adjacent to the area of the pulp chamber of the crown in each experimental tooth, and the compactness of reparative dentin were assessed in longitudinal sections covering the central part of the experimental wound [15]. The areas in the sections were measured using ImageJ software 1.44 (Wayne Rasband, Bethesda, MD, USA) in three sections in each separate tooth (*n* = 16). The formulas for calculating the formation rate and compactness of reparative dentin are given below:

Formation rate (%) = (total area of reparative dentin/coronal pulp chamber area) × 100

Compactness (%) = [1 – (defects area + cells area)/total area of reparative dentin] × 100

Moreover, the degrees of pulp inflammation induced by the materials were represented by four scores as shown in Table 1 [20].

### 2.9. Statistical Analyses

The results from ALP activity assay and alizarin red S staining in vitro as well as the formation rate of reparative dentin and the degrees of pulp inflammation induced by the materials in vivo are expressed as mean ± standard deviation. They were analyzed using a one-way ANOVA test (SPSS-26, IBM, Armonk, New York, NY, USA), the significance level was set at *p* < 0.01 or 0.05.

## 3. Results

### 3.1. Cell Morphology and Proliferation

**Figure 1:**
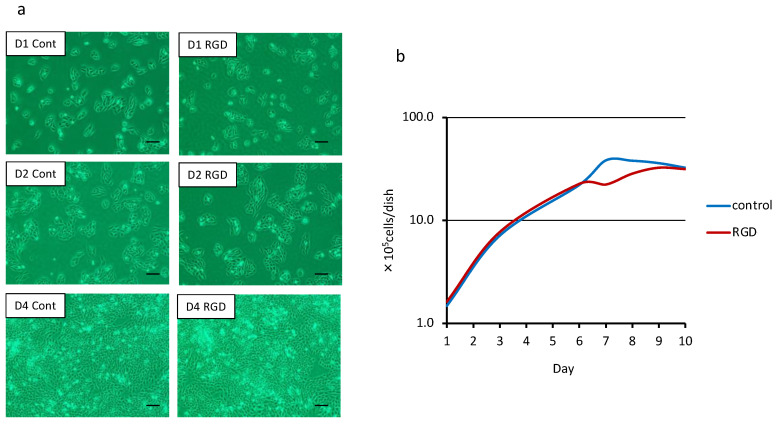
Cell morphology and cell number determination (*n* = 3). MDPC-23 cells were cultured with the DPP-derived RGD-containing peptide. (**a**) No specific morphological changes were observed between the RGD group (RGD) and the control group (Cont) at day 1 (D1), day 2 (D2) and day 4 (D4). Bar = 150 μm. (**b**) Both groups reached confluency on day 4, and cell numbers did not show any difference between the groups either.

Cells were observed on days 1, 2 and 4. After one day, cells displayed an extended morphology in both groups (Figure 1a). The cells in both groups progressively increased in number after two days and reached confluency after four days. No difference was observed between the two groups (Figure 1a) in this respect. Additionally, there was no difference between the two groups regarding cell number in the first 10 days (Figure 1b).

### 3.2. Cell Differentiation and Mineralization

**Figure 2:**
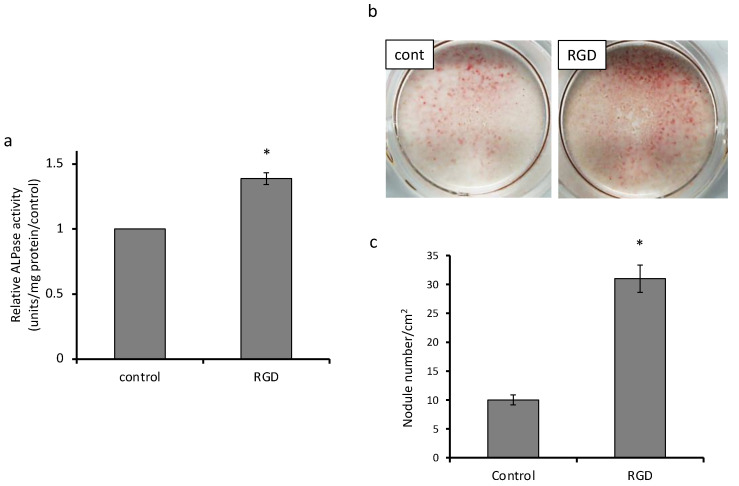
Analysis of differentiation and mineralization (*n* = 3). (**a**) ALP activity of MDPC-23 cells in the experimental group was approximately 1.4 times higher than that in the control group on day 7 (* *p* < 0.01). (**b**) The mineralized nodules were visualized by alizarin red S staining. (**c**) The number of mineralized nodules induced by the DPP-derived RGD-containing peptide rose by approximately three times (* *p* < 0.01).

The most dramatic difference was observed in mineralized nodule formation, which exhibited an approximately six-fold increase as compared to that in the control group (Figure 2c).

ALP activity was measured as a marker of cell differentiation followed by mineralization. ALP activity of MDPC-23 cells in the experimental group was approximately 1.4 times higher (*p* < 0.01) than that in the control group on day 7 (Figure 2a). After alizarin red S staining of MDPC-23 cells on day 8, mineralized nodules were observed in both groups (Figure 2b). Quantitative analyses demonstrated that the number of mineralized nodules in the RGD group was remarkably larger than that in the control group (Figure 2c).

### 3.3. Dentin Regeneration Experiment in Rats

**Figure 3:**
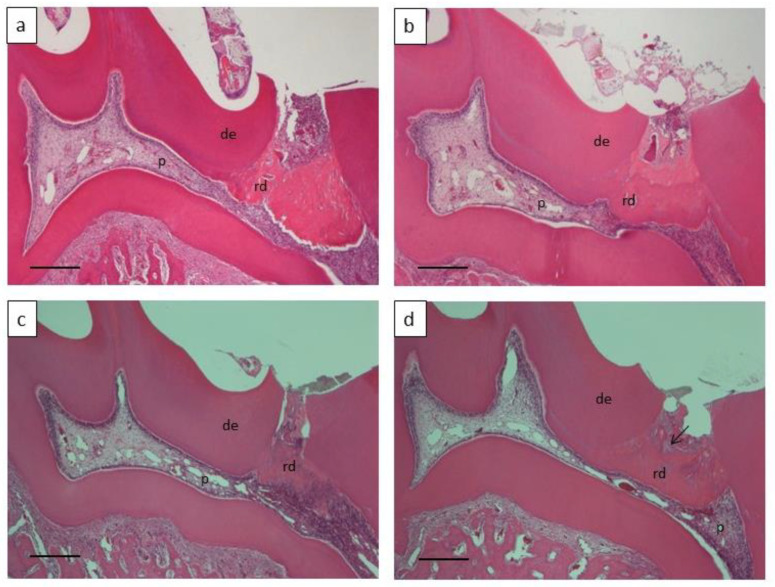
Histological observation of the formed reparative dentin (*n* = 4) treated with (**a**,**b**) the DPP-derived RGD-containing peptide and calcium hydroxide (**c**,**d**). Bar = 150 μm, de: dentin, p: pulp, rd: reparative dentin, arrow: defect.

**Figure 4:**
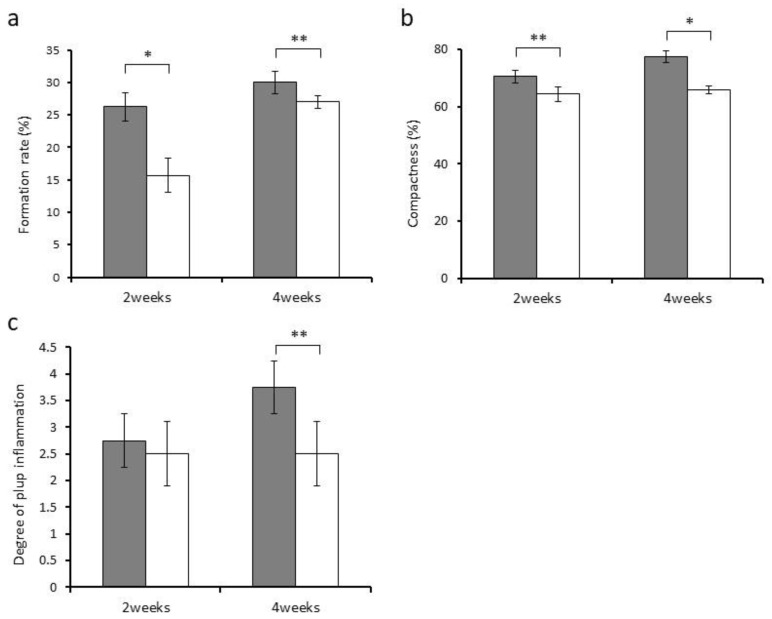
Evaluation of the reparative dentin formed and pulp inflammation triggered by the DPP-derived RGD-containing peptide (gray) and calcium hydroxide (white) after two and four weeks (*n* = 4). (**a**) Formation rate of the reparative dentin (%). (**b**) Compactness of the reparative dentin (%). (**c**) Degree of pulpitis (score). Asterisk indicates significant differences (* *p* < 0.01, ** *p* < 0.05).

We used the DPP-derived RGD-containing peptide and calcium hydroxide for direct pulp capping to compare their effects on reparative dentin formation and pulp inflammation. Histologically, the formed reparative dentin at week 4 turned out to have a porous and rough structure in the Ca group compared with the RGD group (Figure 3d). By contrast, the reparative dentin was observed to have a dense structure in the RGD group (Figure 3b). The DPP-derived RGD-containing peptide was entirely absorbed and replaced with reparative dentin at week 2 (Figure 3a). The morphometrical analysis of the formed reparative dentin demonstrated that the RGD group had a significantly higher formation rate than the Ca group (Figure 4a). Regarding the compactness of the formed reparative dentin, the RGD group exhibited higher values compared with the Ca group (Figure 4b).

Next, we evaluated the degree of pulp inflammation in the sections. The Ca group exhibited pulp inflammation with a capillary expansion of blood vessels and infiltration of inflammatory cells in the coronal pulp tissue at weeks 2 and 4 (Figure 3d). The RGD group also induced slight infiltration of inflammatory cells localized immediately below the reparative dentin formed at week 2 (Figure 3a). Pulp inflammation measurement with the four-score scale described earlier indicated no difference between the groups at week 2. However, the scores at week 4 appeared to be significantly higher (suggesting lower degrees of inflammation) in the RGD group than in the Ca group (Figure 4c).

## 4. Discussion

With increasing life expectancy, people’s demands for medical care have changed from “longevity” to “improvement of quality of life (QOL)”. This has led to the realization of regenerative medicine for various organs and tissues. In particular, hard tissue regeneration is an important issue related to human QOL. In the field of dentistry, tooth loss caused by dental caries, periodontal disease and trauma provokes fundamental oral and general health issues. It is desirable to develop bioengineering techniques for induction of dental tissue and whole-tooth regeneration to restore physiological tooth function. In the present study, we focused on dentin regeneration to restore the function of dental pulp tissue that is essential to maintain a healthy tooth over time. It would be interesting in the future to examine the interaction of dental pulp regeneration compounds with enamel and dentin remineralization to reconstruct the original tooth structure [21,22]. Furthermore, whole-tooth regeneration will eventually be possible by stem cell transplantation, cytokine therapy, cell-sheet technology and so on [23,24,25].

DPP displays various functions under different circumstances. In the present study, we aimed to grasp a comprehensive understanding of both in vitro and in vivo performance of DPP. Some researchers have demonstrated that immobilized DPP induces in vitro mineralization by apatite nuclei formation in a metastable calcium phosphate solution, in vitro odontoblast differentiation in a cell culturing system, and in vivo reparative dentin formation in a direct pulp capping system [12,13,14,15,17,18]. The multifunctionality of immobilized DPP is attributed to its characteristic structure containing repeated Asp-PSer-PSer and cell-binding RGD motifs [8]. Immobilization on some stable support appears to be a primary condition for the performance of DPP. This is because free DPP in solution undergoes aggregation to conceal its active motifs from calcium ions or cell surface receptors; therefore, immobilization of DPP prevents its self-association, thereby allowing it to function effectively [19]. Significantly, the substrate for immobilization functions as a scaffold in drug delivery systems for cell differentiation.

To target odontoblast differentiation by DPP, a DPP-derived RGD-containing peptide consisting of 20 residues was applied in the present study to avoid risks such as virus infection and contamination of infinitesimal unknown components in bio-based raw materials. We observed that the DPP-derived RGD-containing peptide facilitated odontoblast differentiation and mineralization of MDPC-23 cells in vitro. These results can be attributed to the fact that the peptide employed herein—unlike the whole sequence of DPP—does not undergo self-association because it consists of a very short range of amino acid sequences. The results suggest that the proper three-dimensional (3D) conformation of the RGD motif facilitates the peptide to bind with its cell receptors. RGD-containing proteins do not always interact with integrin because the RGD domain may not be localized on their surface [26]. Therefore, the proper 3D conformation of the peptide is essential for its function.

Moreover, the accessibility of RGD domains to integrin is influenced by the flanking amino acid sequences of the RGD domain. Suzuki et al. reported that Ala-Ser next to the RGD domain in DPP is the peptide bond that subjects the domain to sequestration [26]. In the present study, a DPP-derived RGD-containing peptide with an A-S bond promoted cell differentiation. Another sequence indispensable for integrin binding in the peptide or proper configuration of the peptide might play a role in RGD exposure to integrin on cells. Thus, further research is needed to find an appropriate peptide with a better configuration for effectively binding the cell surface integrin and consequently activating cell signaling pathways, such as Mitogen-activated protein kinase (MAPK) signaling pathway [16] and AKT-mTOR signaling pathway [14], thereby leading to odontoblast differentiation.

We confirmed the potential of a DPP-derived RGD-containing peptide to induce odontoblast differentiation in animal experiments. Compared with calcium hydroxide, which has conventionally been used for vital pulp therapy, this peptide provoked intensive dentin regeneration and less pulpitis in rats. Furthermore, the peptide proved to be biocompatible considering that it led to the development of new material without causing chronic inflammation and consequent necrosis of dental pulp tissue. Therefore, it is a strong and safe inducer of dentin regeneration. In this study, we confirmed that calcium hydroxide induced pulpitis and porous reparative dentin formation, which is consistent with previous reports. However, further observation of the long-term pulp vitality is required to judge the excellence of the peptide in vital pulp therapy.

For the effective application of the peptide, a 3D scaffold that properly releases the peptide to ensure that it can migrate toward and attach to target cells and that serves as a substrate for cell proliferation and differentiation (i.e., a drug delivery system) is required [27]. Therefore, the synthesized DPP-derived RGD-containing peptide can be seeded on scaffolds such as collagen (as the major dentin matrix protein), hydroxyapatite (as the main inorganic component of dentin) and polylactic acid (as an artificial biodegradable material) [28,29,30]. By further development of effective application measures of this DPP-derived RGD-containing peptide, it is highly likely to regenerate the entire volume of the dentin lost by dental caries because of its biodegradable property. Therefore, this DPP-derived RGD-containing peptide could potentially be used as a biomimetic material for tooth repair and a tissue engineering therapeutic approach to deep caries treatment.

The present study was limited by its design and data analysis methods. Because dental caries is a bacterial infectious disease, deep caries causes pulp inflammation. Thus, further investigation on the effect of DPP-derived RGD-containing peptide on inflamed pulp tissue and the combined effect of the peptide and anti-inflammatory agent to reduce inflammation at the early healing stage on inflamed pulp tissue are needed using large animals such as rabbits, beagles, etc. Moreover, by 3D analysis, both the quantity and quality of reparative dentin induced by the peptide and the state of pulp tissue will be analyzed more accurately for the judge of the potential of the peptide.

## 5. Conclusions

The current study showed that the DPP-derived RGD-containing peptide SESDNNSSSRGDASYNSDES accelerates in vitro differentiation and mineralization of odontoblasts as well as induces in vivo formation of intensive reparative dentin with high compactness. This peptide is a biocompatible and biodegradable material for dentin regeneration and a promising biomaterial that can be used to formulate new materials for vital pulp therapy.

## Figures and Tables

**Table 1:** The degrees of pulp inflammation induced by the materials.

Degrees of Pulp Inflammation	Score
Minimal inflammation (i.e., no or few scattered inflammatory cells are present in the pulp, similar to the normal dental pulp)	4
Mild inflammation (i.e., a little vasodilation is present, indicating mild hyperemia)	3
Moderate inflammation (i.e., weak vasodilation is present without infiltration of blood cells into the dental pulp, but some inflammatory cells—such as polymorphonuclear leukocytes and neutrophils—can be observed)	2
Severe inflammation (i.e., intense vasodilation is present, appearing as an abscess, and significant inflammatory infiltration of polymorphonuclear leukocytes and neutrophils can be seen throughout the crown)	1

## Data Availability

The data that support the findings of this study are available from the corresponding author upon reasonable request.
